# Design of a standard Iranian protocol of Intravenous thrombolysis with tissue plasminogen activator: A national project

**Published:** 2013

**Authors:** Kavian Ghandehari

**Affiliations:** Professor, Cognitive Research Center, School of Medicine, Mashhad University of Medical Sciences, Mashhad, Iran

**Keywords:** Thrombolysis, Tissue Plasminogen Activator, Iran, Protocol

## Abstract

Standard protocols should be established for treating eligible stroke patients with tissue plasminogen activator (TPA) (recommendation class I, level of evidence B). The Iranian standard protocol of Intravenous Thrombolysis with recombinant Tissue Plasminogen Activator (IVTTPA) is the best possible and easy to use method for performing intravenous thrombolysis in Iran. This protocol overcomes problems and limitations of IVTTPA in Iran. The protocol achieves the best selection criteria and assessment method of IVTTPA for our residents and neurologists. This protocol was provided in Persian language and could be easily downloaded from Google site by writing Thrombolysis and Iran in Persian.

## Introduction

Standard protocols for performing Intravenous Thrombolysis with recombinant Tissue Plasminogen Activator (IVTTPA) are designed in university hospitals and national stroke centers of developed countries.^1^ Executive and medical situation is greatly different in various countries and this affects IVTTPA management.^2^ This difference is huge between developed and developing countries.^2^ For example recombinant tissue plasminogen activator (rTPA) is free of charge for the patients in developed countries, while in developing countries like Iran the patient should be charged 1400 USD which is very expensive.^2^

This situation greatly influences patient selection criteria and cost and effectiveness policy. Therefore, the IVTTPA protocols of developed countries should be modified for using in developing countries like Iran.^2^

## Materials and Methods

***Part I-2009:*** A pilot study for assessment of problems and limitations of IVTTPA in our center was initially performed. This survey included all of the consecutive ischemic stroke patients who were admitted in a university hospital within 3 hours window. Onset to door time, door to needle time, presence of contraindications of IVTTPA, and economic capability of patient to pay for TPA vial were evaluated.^3, 4^

Eight percent of ischemic stroke patients were admitted in hospital within 3 hours window. Forty four percent of this early admitted stroke patients remained within 3 hours window at completed CT-scan and laboratory tests.^3, 4^ Only 36% of these patients had the hospital entrance to completed investigations time less than 60 minutes.^3, 4^ Among stroke patients who admitted to hospital in 3 hours after stroke onset, 42% had no contraindication.^3, 4^ Only 30% of these early arrived stroke patients were capable for payment of TPA expense by themselves. If TPA was free of charge, 86% of early arrived group of our stroke patients missed thrombolysis therapy due to delay in investigations and presence of the contraindications.^3, 4^

The most important barrier of thrombolysis with TPA in Iran is that governmental and health insurance systems do not cover this item for stroke patients.^3, 4^ High cost of rehabilitation therapy and socioeconomic effect of stroke could convince these responsible systems to include TPA in stroke in their insurance coverage. Financial gain of IV thrombolysis with TPA is zero for neurologists based on the governmental and health insurance system rules.^2^ Fear of legal challenges due to intracerebral hemorrhage (ICH) and systemic hemorrhage and other complications makes problem for physician. Private hospitals could easily solve this problem by making internal rules while in governmental hospitals change of the rules is more complex.^2^

***Part II- 2010-2013 to be continued:*** A stroke code was established in our hospital for prevention of delay in triage and investigation of IVTTPA candidates.^1^ A resident selected IVTTPA as thesis and he was directly involved in this management.

We treated eligible patients with IVTTPA in our center based on the American Stroke Association guideline^1^. Excellent recovery, defined as modified Rankin Scale 0-1 at 3 months, was achieved in 62% of the treated patients.^1^ Death due to symptomatic ICH, asymptomatic hemorrhage within infarction and sever brain edema were developed in 14%, 17% and 28% of the treated patients, respectively. 28% of the treated patients had transient bradycardia and 7% of them had transient vomiting during infusion of rTPA. Recurrence of stroke and symptoms due to re-occlusion of the opened artery was found in 10% of the initially successful thrombolysed patients. Learning curves of our residents greatly influenced the results of IVTTPA.

We had 2 deaths due to ICH among our 5 initially treated cases due to protocol violations. Most of the symptomatic ICH and sever brain edema in our patients were developed due to protocol violation. We suggest that IVTTPA should be performed within resident thesis in Iranian university hospitals. This strategy makes 24 hours available neurologist for IVTTPA and prevents delay in door to needle time which is very important for degree of recovery after IVTTPA.^1^ IVTTPA workshops are very effective in increasing knowledge and experience of residents for selection of patients and management.

These workshops were carried out in Shiraz, Ahvaz and Mashhad Universities in 2012-2013. We recommend making an Iranian IVTTPA data bank which registers clinical and imaging data of all IVTTPA patients from all centers in the country. This could make extremely valuable source of research and would improve skills of IVTTPA in our residents and neurologists.

***Part III-2013:*** Influence of problems and limitations of IVTTPA in our center in performing thrombolysis reassessed serially.^2–4^ Methods to overcome these problems in our situation were developed and revised. Numerous standard IVTTPA protocols from North America, Europe and Australia was studied and compared with each other.

Each of our treated patients was evaluated based on the numerous standard IVTTA protocols and eligibility or ineligibility according to each protocol was determined. Result of treatment including recovery and complications of each treated patient based on every standard protocol was evaluated. The standard Iranian IVTTPA was designed to achieve these goals:

1. Minimizing complications of treatment based on improvement of the selection criteria
2. Cost and effectiveness was strictly considered in selection criteria of our IVTTPA protocol due to high cost of TPA vial and not covering TPA by Iranian health insurance companies. This strategy could exclude candidates who probably will not get benefit by IVTTPA or may develop sever complications like symptomatic ICH, sever brain edema or systemic hemorrhage
3. Prevention of protocol violations by clearly defining contraindications of IVTTPA in details
4. Reducing door to needle time by involvement of the resident in bedside of the IVTTPA patient
5. Iranian IVTTPA protocol is a simplified step by step management method for residents of neurology in university hospitals with infrastructure of IVTTPA.

Improvement of learning curve of neurologist and resident is considered in Iranian IVTTPA protocol by detailed illustration of imaging and clinical exclusion criteria. Due to complexity and low inter-rater reliability of NIH stroke scale, modified version of this scale was used in Iranian IVTTPA protocol.^5–7^ This protocol includes practical points in using modified NIH Stroke Scale.^5^

The Alberta Stroke Program Early CT Score (ASPECTS) and Persian Early CT Score (PECTS) were used for improvement of neuroimaging selection of infarcts less than one third of middle cerebral artery territory in the eligible patients.^5, 8^ Illustrations about ASPECTS, PECTS, and posterior circulation ASPECTS as well as a pamphlet was provided in the protocol to improve learning curve of the Iranian residents and neurologists.^8^ The Iranian IVTTPA protocol includes injection and patient care profile with management of complications of IVTTPA.^5^

The Iranian IVTTPA was illustrated in the 20^th^ Iranian Neurology Congress, 2013. This protocol was provided in Persian language and could be easily downloaded from Google site by writing Thrombolysis and Iran in Persian in Google searching engine since 2013. Online access is provided with:^9^ http://www.mums.ac.ir/shares/quaem/eidih1/files/Drghandehari/The%20modified%20NIH%20Stroke%20Scale.pdf?
